# Global, regional, and national burden of visceral leishmaniasis, 1990–2021: findings from the global burden of disease study 2021

**DOI:** 10.1186/s13071-025-06796-x

**Published:** 2025-04-26

**Authors:** Shun-Xian Zhang, Guo-Bing Yang, Jian-Yong Sun, Yong-Jun Li, Jian Yang, Ji-Chun Wang, Yao Deng

**Affiliations:** 1 Longhua Hospital, Shanghai University of Traditional Chinese Medicine, Shanghai, 200032 China; 2 Gansu Provincial Center for Disease Control and Prevention, Lanzhou, 730000 Gansu China; 3 Department of Science and Technology, Chinese Center for Disease Control and Prevention, Beijing, 102206 China; 4 National Health Commission Key Laboratory of Parasitic Disease Control and Prevention, Key Laboratory of Jiangsu Province on Parasite and Vector Control Technology, Jiangsu Institute of Parasitic Diseases, Wuxi, 214064 Jiangsu China

**Keywords:** Visceral leishmaniasis, Global burden of disease, Age-standardized rates

## Abstract

**Background:**

Leishmaniasis is a vector-borne parasitic disease caused by protozoa of the *Leishmania * genus; it is transmitted through the bites of infected phlebotomine sandflies. Clinically, it manifests in three primary forms: cutaneous, mucocutaneous, and visceral leishmaniasis (VL). Among these, VL represents the most severe form, characterized by high morbidity and mortality, and poses a considerable public health burden, particularly in endemic regions. This study utilizes data from the Global Burden of Disease (GBD) study 2021 to conduct a comprehensive analysis of the global epidemiological trends and burden of VL from 1990 to 2021, aiming to generate evidence-based insights to inform prevention and control strategies.

**Methods:**

Using GBD 2021 data, this study examined trends in the incidence, prevalence, mortality, and disability-adjusted life years (DALYs) of VL across 204 countries and territories, stratified by age, sex, and sociodemographic index (SDI) levels. Average annual percent change (AAPC) was calculated to describe trends in age-standardized rates and indicator counts from 1990 to 2021.

**Results:**

From 1990 to 2021, the global age-standardized incidence rate (ASIR; AAPC = −0.25, 95% confidence interval (CI) −0.25, −0.24), age-standardized prevalence rate (ASPR; AAPC = −0.06, 95% CI −0.06, −0.05), age-standardized mortality rate (ASMR; AAPC = −0.03, 95% CI −0.04, −0.02), and DALY rate (AAPC = −2.38, 95% CI −2.44, −2.33) for VL all showed a declining trend. The ASMR was highest among children under 5 years old and decreased progressively with age. VL remains a critical and under-recognized tropical disease in Latin America, the Middle East, Africa, and South Asia.

**Conclusions:**

VL disproportionately affects males and presents the highest risk in children under 5 years. Enhanced global collaboration in infectious disease control, with a focus on regions such as Latin America, Africa, the Middle East, and South Asia, is essential to further reduce the burden of VL.

**Graphical abstract:**

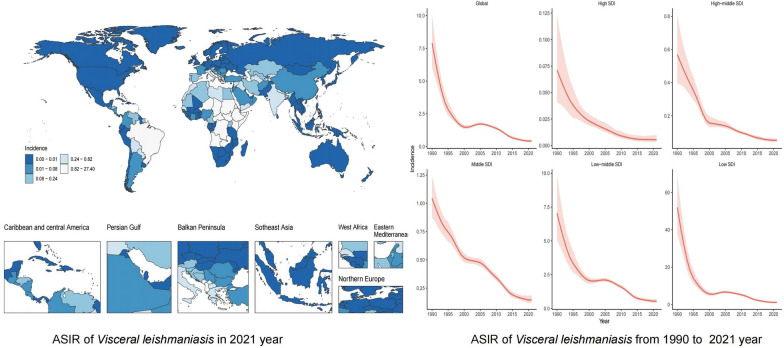

**Supplementary Information:**

The online version contains supplementary material available at 10.1186/s13071-025-06796-x.

## Background

Leishmaniasis, also known as kala-azar, is a parasitic disease caused by protozoan parasites of the *Leishmania* genus, which are transmitted to humans through the bite of infected female phlebotomine sandflies, including visceral leishmaniasis (VL), cutaneous leishmaniasis, and mucocutaneous leishmaniasis [1–4]. VL is a disease caused by two known vector-borne parasite species (*Leishmania donovani* and *Leishmania infantum*) transmitted to man by phlebotomine sandflies (species: *Phlebotomus* and *Lutzomyia*) [5–7]. VL affects critical organs and tissues such as the spleen, liver, and bone marrow, leading to symptoms such as recurrent fever, weight loss, hepatosplenomegaly, and anemia. Without timely treatment, VL has a mortality rate exceeding 95% [3–5].

Although the global incidence of VL has shown a declining trend in recent years, VL remains one of the key neglected tropical diseases prioritized by the World Health Organization (WHO), with a high mortality and morbidity burden. The disease burden is predominantly concentrated in South Asia, East Africa, the Mediterranean region, South America, and Central Asia [8–11]. However, shifts in epidemiological dynamics have hindered the decline in VL incidence. Notably, human immunodeficiency virus (HIV) has increased the complexity of treatment and led to higher relapse rates for HIV–VL co-infection [12]. In addition, urbanization, global warming, and changing precipitation patterns have expanded the ecological niche of sandflies, potentially contributing to the emergence of VL in nonendemic areas and an increase in cases in traditionally endemic regions [13–15].

Since the 1990s, multiple studies have attempted to estimate the global burden of VL, revealing substantial variations in VL incidence and disability-adjusted life years (DALYs) across different regions [4, 9, 16]. Moreover, the temporal trends in VL burden differ across countries, highlighting the need for a comprehensive assessment of its global distribution and trajectory. Therefore, to comprehensively understand the burden and trends of VL across countries worldwide, we utilized data from the Global Burden of Disease (GBD) study 2021 [17, 18], which provides estimates of VL burden across 204 countries and territories from 1990 to 2021. Understanding the global trends in VL burden is crucial for informing targeted interventions, optimizing policy decisions, allocating resources efficiently, strengthening global health collaboration, and advancing VL elimination efforts.

## Methods

### Date source

The data for this study were derived from the GBD 2021 database (https://vizhub.healthdata.org/gbd-results); it provides high-quality estimates of key epidemiological indicators for 371 diseases and injuries across 204 countries and territories [17–20]. The GBD study employs a range of sophisticated methodologies to integrate data from various sources, including mortality and incidence registries, surveys, and systematic literature reviews. In addition, GBD 2021 offers an analysis of risk factors, providing comprehensive estimates of exposure levels, relative health risks, and attributable disease burden for 88 risk factors across 204 countries and territories, as well as 811 subnational locations, from 1990 to 2021. Detailed descriptions of the study design, data collection, and estimation methods are available in published literature [17–20].

The GBD study 2021 model incorporated cases classified under the International Classification of Diseases (ICD) for VL, specifically ICD-9 code 085.0 and ICD-10 code B55.0 [17]. The study analyzed data on incidence, prevalence, mortality, and disability-adjusted life-years (DALYs) for VL from 1990 to 2021, stratified by sex (male, female, and both combined) and specific age groups. Estimates were provided at the global level, across five sociodemographic index (SDI) regions, 21 geographical regions, and 204 countries and territories. In addition, age-standardized rates and absolute case numbers for four metrics (incidence, prevalence, death, and DALYs) were assessed for all-age groups. Data were sourced from the Global Health Data Exchange query tool [17].

The SDI, used in the GBD Study 2021 database, is a composite measure of overall socioeconomic development [17]. The SDI is calculated on the basis of educational attainment among individuals aged 15 years and older, lag-distributed income per capita, and the total fertility rate in females under the age of 25 years. Countries and territories are classified into different development levels on the basis of the following SDI thresholds: low SDI regions (0–0.4658), low-middle SDI regions (0.4658–0.6188), middle SDI regions (0.6188–0.7120), high-middle SDI regions (0.7120–0.8103), and high SDI regions (0.8103–1.0000). This categorization allows for a more systematic analysis of the impact of socioeconomic development levels on health outcomes [17].

### Statistical analysis

The disease burden of VL was assessed using rates and total case counts for incidence, prevalence, mortality, and DALYs. The rate was expressed as estimates per 100,000 population, reflecting the relative burden, while case counts represented the absolute burden. Both metrics are reported with 95% uncertainty intervals (UI). Consequently, when comparing two numerical values (numbers, rates, or percentages), statistical significance could not be directly calculated. If the UIs or confidence intervals (CIs) overlapped, it indicated no significant difference (*P* > 0.05). Conversely, if the UIs or CIs did not overlap, a statistical difference existed (*P* < 0.05) [21]. In addition, case fatality rates were used to assess the mortality risk across different age groups [22].

All statistical analyses were conducted using R software (version 4.4.1, R Foundation for Statistical Computing, Vienna, Austria; available at https://cran.r-project.org). Detailed descriptions of specific analytical methods are provided [23–26].

### Joinpoint regression analysis

The average annual percentage change (AAPC) and 95% confidence intervals (CIs) were used as key indicators to analyze the trends in age-standardized rate (ASR, including age-standardized incidence rate (ASIR), age-standardized prevalence rate (ASPR), age-standardized mortality rate (ASMR), and age-standardized DALY rate) for VL from 1990 to 2021. The AAPC model applied segmented regression to the log-linear equation ln(*y*) = *β* × year + constant, to identify inflection points in the trends. A grid search method was used to calculate all possible breakpoints, selecting the one with the minimum mean squared error as the optimal breakpoint [23]. The number of optimal breakpoints was further determined through a Monte Carlo permutation test, allowing for a range of 0–5 breakpoints. The calculation is as follows [23, 27, 28]:

$${\text{APC}}_{i} = \left\{ {\exp \left( {\beta_{i} } \right) - 1} \right\} \times 100$$

$${\text{AAPC}}_{i} = \left\{ {\exp \left( {\frac{{\sum {W_{i} \beta_{i} } }}{{W_{i} }}} \right) - 1} \right\} \times 100$$

In the model, *i* is the number of segments, $\beta_{i}$ is the regression coefficient from the log-linear model ln(*y*) = *β* × year + constant. $W_{i}$ represents the length of each corresponding segment. When the AAPC is greater than 0 with a *P* value less than 0.05, it indicates a statistically significant upward trend. Conversely, an AAPC less than 0 with a *P* value below 0.05 denotes a statistically significant downward trend. The AAPC reflects the overall trend by weighting the APC for each segment on the basis of the duration of the respective time spans. This analytical approach not only improves the precision of trend identification over time but also enhances the robustness of the model [23].

### Estimated annual percentage change (EAPC)

To assess the temporal trends of VL from 1990 to 2021, this study used the EAPC to quantify changes in disease burden metrics, including ASIR, ASPR, ASMR, and age-standardized DALY rate. The EAPC was calculated by fitting a log-linear regression model [21, 23, 29, 30]:

$$y = \alpha + \beta x + \varepsilon$$

where *y* is equal to natural logarithm of (rate), *x* signifies the calendar year, and *ε* denotes an independent, normally distributed error term. An EAPC value less than 0, with the upper bound of its 95% CI also below 0, indicates a declining trend. Conversely, an EAPC value greater than 0, with the lower bound of its 95% CI above 0, signifies an increasing trend [21, 23].

### Association between SDI and ARS

Smoothing spline models were utilized to examine the relationship between ASIR, ASPR, and age-standardized DALY rate of VL, as well as the SDI across 21 GBD regions and 204 countries and territories. The smoothing splines were constructed using the locally weighted scatterplot smoothing method, which adaptively determines the degree, number, and placement of knots on the basis of the data distribution and span parameter [29, 31, 32]. In addition, Spearman’s correlation analysis was performed to calculate the correlation coefficients (*R* indices) and corresponding *P* values to assess the association between ASR and SDI, with statistical significance set at *P* < 0.05 [29, 31, 32].

## Results

### Global

In 2021, the global ASIR, ASPR, ASMR, and age-standardized DALY rate for VL were 0.42 per 100,000 population (95% UI: 0.34, 0.53), 0.10 per 100,000 population (95% UI: 0.08, 0.13), 0.07 per 100,000 population (95% UI: 0.02, 0.24), and 5.39 per 100,000 population (95% UI: 1.70, 17.12), respectively. From 1990 to 2021, the ASIR (AAPC = −0.25, 95% CI −0.25, −0.24), ASPR (AAPC = −0.06, 95% CI −0.06, −0.05), ASMR (AAPC = −0.03, 95% CI −0.04, −0.02), and age-standardized DALY rate also showed a marked decline (AAPC = −2.38, 95% CI −2.44, −2.33) in VL. Concurrently, the absolute numbers of VL cases, including incidence, prevalence, and deaths, demonstrated a downward trend (Additional File 1: Supplementary Tables S1–S3). However, the total number of DALY cases attributable to VL showed an increasing trend (Additional File 1: Supplementary Table S4).

### Five SDI regions

**Fig. 1:**
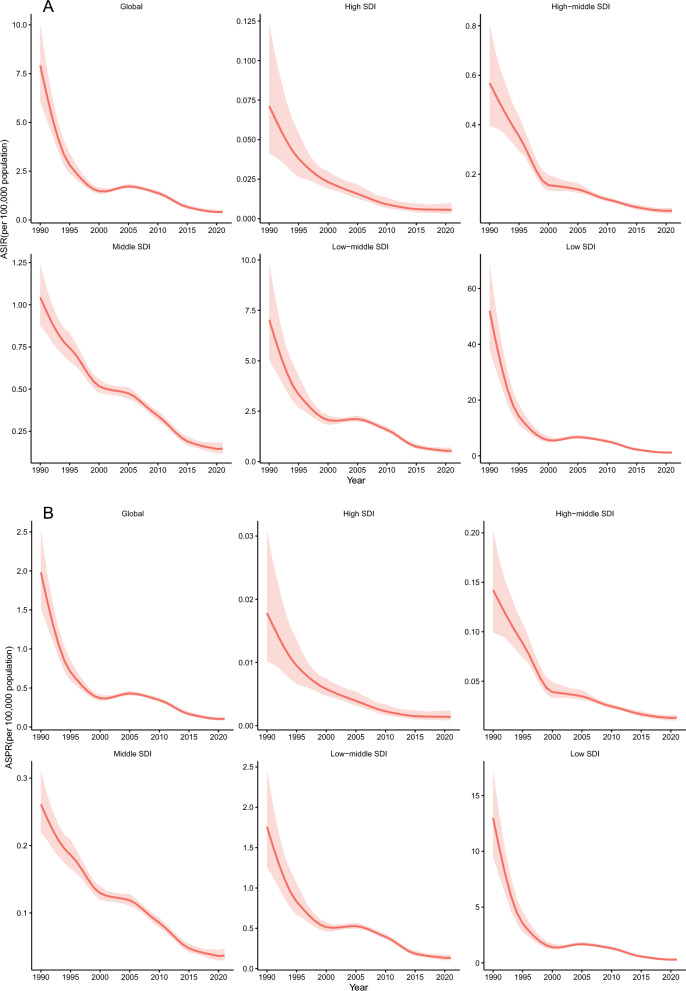

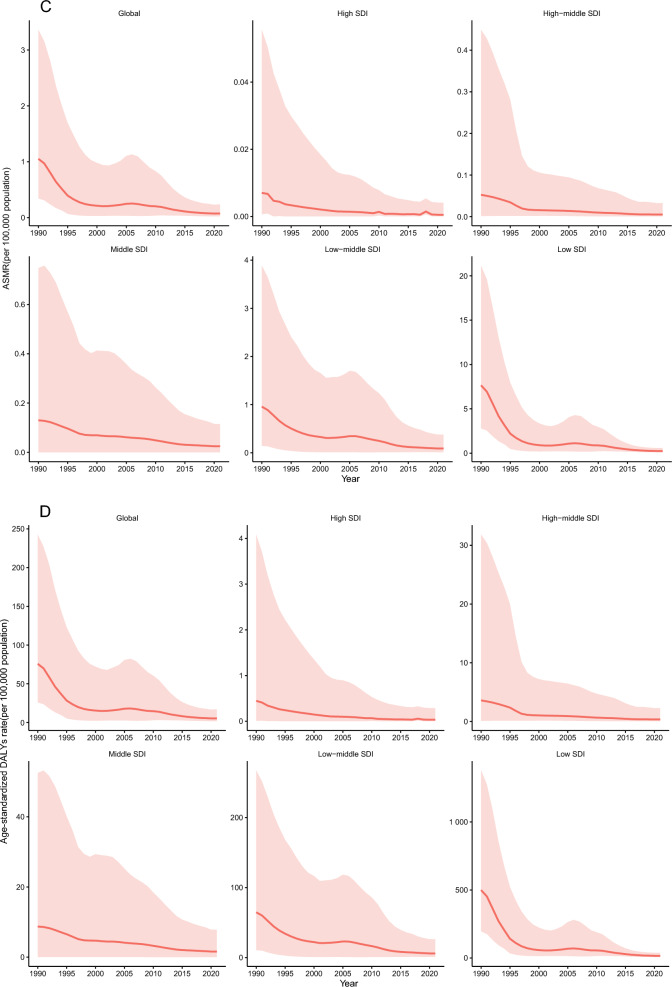
The changing trends of age-standardized rates for visceral leishmaniasis were analyzed among the global population and five SDI regions (**A** ASIR; **B** ASPR; **C** ASMR; **D** age-standardized DALY rate) *ASIR* age-standardized incidence rate, *ASMR* age-standardized mortality rate, *ASPR* age-standardized prevalence rate, *DALYs* disability-adjusted life years, *SDI* sociodemographic index

In 2021, the ASIR, ASPR, ASMR, and age-standardized DALY rate of VL were highest in low SDI regions and lowest in high SDI regions. From 1990 to 2021, all four metrics—ASIR, ASPR, ASMR, and age-standardized DALY rate—declined across the five SDI levels, with the most pronounced reductions observed in low SDI regions (Tables 1, 2, 3, 4; Fig. 1A–D).

**Table 1:** The ASIR of VL in the years 1990 and 2021 and change trend of ASIR were analyzed across GBD regions

Location	ASIR (per 100,000 population, 95% UI) 1990	ASIR (per 100,000 population, 95% UI) 2021	Percentage change (95% UI) 1990–2021	AAPC (95% CI) 1990–2021
Global	7.92 (6.05, 10.07)	0.42 (0.34, 0.53)	−94.75 (−96.08, −92.36)	−0.25 (−0.25, −0.24)
East Asia	0.43 (0.31, 0.60)	0.07 (0.06, 0.10)	−82.55 (−88.60, −73.10)	−0.01 (−0.01, −0.00)
Southeast Asia	0.01 (0.00, 0.01)	0.00 (0.00, 0.00)	−85.10 (−93.44, −62.76)	−0.01 (−0.01, −0.00)
Central Asia	0.27 (0.19, 0.40)	0.41 (0.30, 0.60)	53.44 (−6.37, 150.48)	0.01 (0.00, 0.01)
Central Europe	1.47 (0.82, 2.98)	0.08 (0.06, 0.12)	−94.53 (−97.50, −88.63)	−0.05 (−0.05, −0.04)
Western Europe	0.24 (0.18, 0.30)	0.08 (0.05, 0.12)	−66.32 (−79.35, −47.15)	−0.01 (−0.01, −0.00)
Southern Latin America	0.08 (0.03, 0.14)	0.02 (0.01, 0.03)	−75.09 (−90.31, −36.85)	−0.01 (−0.01, −0.00)
Caribbean	0.03 (0.00, 0.08)	0.00 (0.00, 0.01)	−91.63 (−98.75, −46.43)	−0.01 (−0.01, −0.00)
Andean Latin America	0.85 (0.15, 2.84)	0.05 (0.01, 0.15)	−94.58 (−99.31, −60.84)	−0.03 (−0.03, −0.02)
Central Latin America	0.18 (0.12, 0.27)	0.05 (0.04, 0.07)	−72.27 (−83.32, −56.12)	−0.01 (−0.01, −0.00)
Tropical Latin America	3.03 (2.28, 3.93)	2.55 (1.49, 4.07)	−16.06 (−52.91, 42.88)	−0.02 (−0.02, −0.01)
North Africa and the Middle East	9.82 (6.66, 14.30)	0.71 (0.44, 1.11)	−92.80 (−96.11, −86.97)	−0.30 (−0.31, −0.29)
South Asia	19.36 (13.11, 27.24)	0.38 (0.21, 0.64)	−98.01 (−98.97, −96.13)	−0.63 (−0.65, −0.61)
Central Sub-Saharan Africa	33.03 (11.18, 82.91)	1.96 (0.61, 4.95)	−94.07 (−98.66, −75.76)	−0.98 (−1.00, −0.96)
Eastern Sub-Saharan Africa	50.22 (30.32, 78.53)	1.41 (1.08, 1.85)	−97.19 (−98.33, −94.95)	−1.56 (−1.59, −1.53)
Western Sub-Saharan Africa	0.05 (0.03, 0.08)	0.17 (0.07, 0.39)	264.97 (29.99, 948.47)	0.01 (0.00, 0.01)
High SDI	0.07 (0.04, 0.12)	0.01 (0.00, 0.01)	−92.14 (−96.24, −82.78)	−0.01 (−0.01, −0.00)
High-middle SDI	0.57 (0.40, 0.81)	0.05 (0.04, 0.07)	−90.78 (−93.78, −85.80)	−0.02 (−0.02, −0.01)
Middle SDI	1.04 (0.87, 1.24)	0.15 (0.12, 0.19)	−85.99 (−89.43, −81.13)	−0.03 (−0.03, −0.02)
Low-middle SDI	7.04 (5.04, 9.82)	0.54 (0.40, 0.70)	−92.34 (−95.07, −87.98)	−0.20 (−0.21, −0.19)
Low SDI	52.00 (37.84, 68.97)	1.17 (0.84, 1.68)	−97.76 (−98.57, −96.42)	−1.63 (−1.70, −1.55)

The value “0.00” does not represent an absolute zero; rather, the actual value contains nonzero digits beyond the second decimal place, but owing to rounding (following the round half-up rule), it appears as “0.00” when displayed with two decimal places. In the GBD 2021 database, 204 countries and territories are categorized into 21 geographical regions. However, no data on the ASIR of VL were available for Oceania, Eastern Europe, high-income North America, high-income Asia Pacific, Australasia, or Southern Sub-Saharan Africa; *AAPC* average annual percent change, *ASIR* age-standardized incidence rate, *CI* confidence interval, *GBD* Global Burden of Disease, *SDI* sociodemographic index, *UI* uncertainty interval, *VL* visceral leishmaniasis

**Table 2:** The ASPR of VL in the years 1990 and 2021 and change trend of ASPR were analyzed across GBD regions

Location	ASPR (per 100,000 population, 95% UI) 1990	ASPR (per 100,000 population, 95% UI) 2021	Percentage change (95% UI) 1990–2021	AAPC (95% CI) 1990–2021
Global	1.98 (1.51, 2.52)	0.10 (0.08, 0.13)	−94.75 (−96.08, −92.36)	−0.06 (−0.06, −0.05)
East Asia	0.11 (0.08, 0.15)	0.02 (0.01, 0.02)	−82.55 (−88.60, −73.10)	−0.00 (−0.01, −0.00)
Southeast Asia	0.01 (0.00, 0.01)	0.00 (0.00, 0.01)	−85.10 (−93.44, −62.76)	−0.00 (−0.01, −0.00)
Central Asia	0.07 (0.05, 0.10)	0.10 (0.07, 0.15)	53.44 (−6.37, 150.48)	0.01 (0.01, 0.01)
Central Europe	0.37 (0.20, 0.75)	0.02 (0.01, 0.03)	−94.53 (−97.50, −88.63)	−0.01 (−0.01, −0.00)
Western Europe	0.06 (0.05, 0.08)	0.02 (0.01, 0.03)	−66.32 (−79.35, −47.15)	−0.01 (−0.01, −0.00)
Southern Latin America	0.02 (0.01, 0.04)	0.00 (0.00, 0.01)	−75.09 (−90.31, −36.85)	−0.01 (−0.01, −0.00)
Caribbean	0.01 (0.00, 0.02)	0.00 (0.00, 0.01)	−91.63 (−98.75, −46.43)	−0.01 (−0.01, −0.00)
Andean Latin America	0.21 (0.04, 0.71)	0.01 (0.00, 0.04)	−94.58 (−99.31, −60.84)	−0.01 (−0.01, −0.00)
Central Latin America	0.05 (0.03, 0.07)	0.01 (0.01, 0.02)	−72.27 (−83.32, −56.12)	−0.01 (−0.01, −0.00)
Tropical Latin America	0.76 (0.57, 0.98)	0.64 (0.37, 1.02)	−16.06 (−52.91, 42.88)	−0.01 (−0.01, −0.00)
North Africa and the Middle East	2.45 (1.66, 3.58)	0.18 (0.11, 0.28)	−92.80 (−96.11, −86.97)	−0.07 (−0.08, −0.07)
South Asia	4.84 (3.28, 6.81)	0.10 (0.05, 0.16)	−98.01 (−98.97, −96.13)	−0.16 (−0.16, −0.15)
Central Sub-Saharan Africa	8.26 (2.80, 20.73)	0.49 (0.15, 1.24)	−94.07 (−98.66, −75.76)	−0.25 (−0.25, −0.24)
Eastern Sub-Saharan Africa	12.55 (7.58, 19.63)	0.35 (0.27, 0.46)	−97.19 (−98.33, −94.95)	−0.39 (−0.40, −0.38)
Western Sub-Saharan Africa	0.01 (0.01, 0.02)	0.04 (0.02, 0.10)	264.97 (29.99, 948.47)	0.01 (0.01, 0.00)
High SDI	0.02 (0.01, 0.03)	0.00 (0.00, 0.01)	−92.14 (−96.24, −82.78)	−0.01 (−0.01, −0.00)
High-middle SDI	0.14 (0.10, 0.20)	0.01 (0.01, 0.02)	−90.78 (−93.78, −85.80)	−0.00 (−0.01, −0.00)
Middle SDI	0.26 (0.22, 0.31)	0.04 (0.03, 0.05)	−85.99 (−89.43, −81.13)	−0.01 (−0.01, −0.00)
Low-middle SDI	1.76 (1.26, 2.46)	0.13 (0.10, 0.18)	−92.34 (−95.07, −87.98)	−0.05 (−0.05, −0.04)
Low SDI	13.00 (9.46, 17.24)	0.29 (0.21, 0.42)	−97.76 (−98.57, −96.42)	−0.41 (−0.42, −0.40)

The value “0.00” does not represent an absolute zero; rather, the actual value contains nonzero digits beyond the second decimal place, but owing to rounding (following the round half-up rule), it appears as “0.00” when displayed with two decimal places. In the GBD 2021 database, the world’s 204 countries and territories are grouped into 21 geographical regions. However, no data on the ASPR of VL were available for Oceania, Eastern Europe, high-income North America, high-income Asia Pacific, Australasia, and Southern Sub-Saharan Africa; *AAPC* average annual percent change, *ASPR* age-standardized prevalence rate, *CI* confidence interval, *GBD* Global Burden of Disease, *SDI* sociodemographic index, *UI* uncertainty interval, *VL* visceral leishmaniasis

**Table 3:** The ASMR of VL in the years 1990 and 2021 and change trend of ASMR were analyzed across GBD regions

Location	ASMR (per 100,000 population, 95% UI) 1990	ASMR (per 100,000 population, 95% UI) 2021	Percentage change (95% UI) 1990–2021	AAPC (95% CI) 1990–2021
Global	1.05 (0.34, 3.36)	0.07 (0.02, 0.24)	−92.93 (−94.26, −90.65)	−0.03 (−0.04, −0.02)
East Asia	0.07 (0.00, 0.39)	0.01 (0.00, 0.06)	−84.43 (−87.36, −80.45)	−0.01 (−0.01, −0.00)
Southeast Asia	0.00 (0.00, 0.01)	0.00 (0.00, 0.01)	−83.40 (−85.96, −76.88)	−0.01 (−0.01, −0.00)
Central Asia	0.02 (0.00, 0.21)	0.04 (0.00, 0.32)	52.03 (38.30, 76.83)	0.01 (0.00, 0.01)
Central Europe	0.12 (0.00, 1.15)	0.01 (0.00, 0.05)	−95.29 (−96.08, −94.36)	−0.01 (−0.01, −0.00)
Western Europe	0.02 (0.01, 0.15)	0.01 (0.00, 0.04)	−71.68 (−74.91, −60.47)	−0.01 (−0.01, −0.00)
Southern Latin America	0.01 (0.00, 0.06)	0.00 (0.00, 0.01)	−74.55 (−76.71, −72.75)	−0.01 (−0.01, −0.00)
Caribbean	0.00 (0.00, 0.02)	0.00 (0.00, 0.01)	−90.81 (−92.91, −87.75)	−0.01 (−0.01, −0.00)
Andean Latin America	0.18 (0.00, 0.86)	0.01 (0.00, 0.05)	−94.35 (−95.82, −92.57)	−0.01 (−0.01, −0.00)
Central Latin America	0.02 (0.00, 0.16)	0.01 (0.00, 0.04)	−71.49 (−76.71, −62.45)	−0.01 (−0.01, −0.00)
Tropical Latin America	0.66 (0.00, 2.44)	0.51 (0.00, 1.80)	−23.28 (−30.61, −4.30)	−0.01 (−0.01, −0.00)
North Africa and the Middle East	0.86 (0.00, 6.88)	0.07 (0.00, 0.56)	−92.36 (−94.51, −90.96)	−0.03 (−0.03, −0.02)
South Asia	2.37 (0.00, 10.32)	0.07 (0.00, 0.35)	−97.06 (−98.03, −96.49)	−0.08 (−0.08, −0.07)
Central Sub-Saharan Africa	5.51 (2.92, 8.70)	0.37 (0.19, 0.61)	−93.24 (−94.43, −91.86)	−0.17 (−0.17, −0.16)
Eastern Sub-Saharan Africa	9.40 (6.40, 12.93)	0.31 (0.19, 0.47)	−96.66 (−97.51, −95.68)	−0.31 (−0.32, −0.30)
Western Sub-Saharan Africa	0.01 (0.01, 0.02)	0.04 (0.02, 0.06)	312.44 (230.37, 412.62)	0.01 (0.01, 0.02)
High SDI	0.01 (0.00, 0.06)	0.00 (0.00, 0.01)	−93.01 (−99.26, −91.20)	−0.01 (−0.01, −0.00)
High-middle SDI	0.05 (0.00, 0.45)	0.01 (0.00, 0.03)	−90.10 (−92.73, −50.61)	−0.01 (−0.01, −0.00)
Middle SDI	0.13 (0.00, 0.75)	0.02 (0.00, 0.11)	−81.02 (−84.68, −42.02)	−0.01 (−0.01, −0.00)
Low-middle SDI	0.96 (0.14, 3.89)	0.09 (0.01, 0.38)	−90.63 (−92.66, −88.63)	−0.03 (−0.03, −0.02)
Low SDI	7.66 (2.79, 21.15)	0.25 (0.10,0.59)	−96.80 (−97.64, −94.87)	−0.24 (−0.26, −0.24)

The value “0.00” does not represent an absolute zero; rather, the actual value contains nonzero digits beyond the second decimal place, but owing to rounding (following the round half-up rule), it appears as “0.00” when displayed with two decimal places. In the GBD 2021 database, the world’s 204 countries and territories are categorized into 21 geographical regions. Among these, no data on the ASMR of VL were available for Oceania, Eastern Europe, high-income North America, high-income Asia Pacific, Australasia, and Southern Sub-Saharan Africa; *AAPC* average annual percent change, *ASMR* age-standardized mortality rate, *CI* confidence interval, *GBD* Global Burden of Disease, *SDI* sociodemographic index, *UI* uncertainty interval, *VL* visceral leishmaniasis

**Table 4:** The age-standardized DALY rate of VL in the years 1990 and 2021 and change trend of age-standardized DALY rate were analyzed across GBD regions

Location	Age-standardized DALY rate (per 100,000 population, 95% UI) 1990	Age-standardized DALY rate (per 100,000 population, 95% UI) 2021	Percentage change (95% UI) 1990–2021	AAPC (95% CI) 1990–2021
Global	75.73 (25.98, 242.77)	5.39 (1.70, 17.12)	−92.89 (−94.30, −90.41)	−2.38 (−2.44, −2.33)
East Asia	5.05 (0.01, 28.05)	0.75 (0.01, 4.31)	−85.17 (−88.30, −80.20)	−0.14 (−0.14, −0.14)
Southeast Asia	0.10 (0.01, 0.45)	0.02 (0.01, 0.07)	−84.51 (−87.16, −78.55)	−0.01 (−0.01, −0.00)
Central Asia	1.58 (0.01, 14.46)	2.40 (0.01,21.49)	52.33 (25.52, 100.08)	0.03 (0.02, 0.04)
Central Europe	8.40 (0.02, 80.50)	0.39 (0.00, 3.75)	−95.35 (−96.46, −92.90)	−0.26 (−0.26, −0.25)
Western Europe	1.51 (0.31, 10.69)	0.44 (0.13, 2.67)	−70.72 (−75.45, −54.28)	−0.04 (−0.04, −0.03)
Southern Latin America	0.38 (0.00, 3.97)	0.10 (0.00, 1.02)	−74.07 (−83.83, −56.51)	−0.01 (−0.01, −0.01)
Caribbean	0.32 (0.01, 1.56)	0.03 (0.01, 0.14)	−90.77 (−94.14, −84.11)	−0.01 (−0.02, −0.01)
Andean Latin America	12.27 (0.01, 62.51)	0.70 (0.01, 3.50)	−94.33 (−96.36, −90.79)	−0.37 (−0.38, −0.37)
Central Latin America	1.65 (0.01, 11.40)	0.47 (0.01, 3.15)	−71.39 (−78.68, −61.53)	−0.04 (−0.04, −0.03)
Tropical Latin America	45.39 (0.06, 173.81)	33.81 (0.06, 124.09)	−25.51 (−35.24, 0.43)	−0.38 (−0.51, −0.26)
North Africa and the Middle East	55.93 (0.16, 469.65)	4.64 (0.01, 40.60)	−91.70 (−94.45, −89.50)	−1.75 (−1.80, −1.71)
South Asia	151.80 (0.33, 692.02)	4.70 (0.01, 24.71)	−96.91 (−98.26, −96.28)	−4.75 (−4.97, −4.53)
Central Sub-Saharan Africa	388.21 (203.34, 616.68)	25.81 (13.13, 41.98)	−93.35 (−94.51, −92.02)	−11.86 (−12.20, −11.52)
Eastern Sub-Saharan Africa	652.48 (441.07, 908.18)	22.32 (13.23, 33.92)	−96.58 (−97.48, −95.55)	−21.20 (−21.85, −20.54)
Western Sub-Saharan Africa	0.69 (0.38, 1.12)	2.89 (1.50, 4.78)	317.59 (235.31, 420.75)	0.07 (0.07, 0.08)
High SDI	0.45 (0.01, 4.09)	0.03 (0.01, 0.29)	−92.68 (−94.65, −87.07)	−0.01 (−0.02, −0.01)
High-middle SDI	3.58 (0.09, 31.85)	0.35 (0.04, 2.27)	−90.36 (−92.91, −54.13)	−0.11 (−0.11, −0.10)
Middle SDI	8.72 (0.02, 52.49)	1.59 (0.01, 7.81)	−81.78 (−85.17, −67.58)	−0.24 (−0.25, −0.24)
Low-middle SDI	64.74 (10.67, 267.27)	6.00 (0.93, 26.29)	−90.73 (−92.51, −88.67)	−1.90 (−1.97, −1.82)
Low SDI	499.55 (195.85, 1379.04)	16.41 (6.87, 38.70)	−96.72 (−97.64, −94.73)	−16.16 (−16.50, −15.81)

The value “0.00” does not represent an absolute zero; rather, the actual value contains nonzero digits beyond the second decimal place, but owing to rounding (following the round half-up rule), it appears as “0.00” when displayed with two decimal places. In the GBD 2021 database, the world’s 204 countries and territories are classified into 21 geographical regions. However, no data on the age-standardized DALYs rate for visceral VL were available for Oceania, Eastern Europe, high-income North America, high-income Asia Pacific, Australasia, and Southern Sub-Saharan Africa; *AAPC* average annual percent change, *CI* confidence interval, *GBD* Global Burden of Disease, *SDI* sociodemographic index, *UI* uncertainty interval, *VL* visceral leishmaniasis

### Geographical regions

In 2021, ASIR, ASPR, ASMR, and age-standardized DALY rate for VL were not reported in six geographic regions: Oceania, Eastern Europe, high-income Asia Pacific, Australasia, high-income North America, and Southern Sub-Saharan Africa. Only 15 geographic regions reported these VL metrics. Among these regions, Tropical Latin America recorded the highest ASIR, ASPR, ASMR, and age-standardized DALY rate for VL in 2021. From 1990 to 2021, ASIR, ASPR, ASMR, and age-standardized DALY rates of VL increased in Central Asia and Western Sub-Saharan Africa. In contrast, these metrics declined in 13 regions, with the most substantial decrease observed in Eastern Sub-Saharan Africa, followed by Central Sub-Saharan Africa and South Asia (Tables 1, 2, 3, 4).

### Countries and territories

In 2021, 84 countries reported the ASIR for VL, with South Sudan recording the highest ASIR at 27.13 per 100,000 population (95% UI: 15.47, 45.99). From 1990 to 2021, ASIR increased in 16 countries, with the largest rise observed in Monaco (AAPC = 0.15, 95% CI 0.15, 0.16). Conversely, ASIR declined in 65 countries, with the steepest reduction seen in Somalia (AAPC = −10.70, 95% CI −10.85, −10.55. Additional File 1: Supplementary Table S5).

Similarly, in 2021, 84 countries reported the ASPR for VL, with South Sudan also showing the highest ASPR at 6.78 per 100,000 population (95% UI: 3.87, 11.50). From 1990 to 2021, ASPR increased in 16 countries, with Monaco again experiencing the largest rise (AAPC = 0.04, 95% CI 0.04, 0.05). In contrast, ASPR decreased in 66 countries, with Somalia showing the greatest decline (AAPC = −2.67, 95% CI −2.71, −2.63. Additional File 1: Supplementary Table S5).

In 2021, 84 countries reported the ASMR for VL, with the highest ASMR observed in the Republic of South Sudan at 6.13 per 100,000 population (95% UI: 3.18, 19.92). Between 1990 and 2021, ASMR increased in 17 countries, with the greatest rise recorded in Monaco (AAPC = 0.01, 95% CI: 0.01, 0.02). Conversely, ASMR declined in 65 countries, with the steepest decrease noted in South Sudan (AAPC = −2.02, 95% CI −2.27, −1.76. Additional File 1: Supplementary Table S5).

Similarly, in 2021, 84 countries reported the age-standardized DALY rate for VL, with South Sudan again recording the highest rate at 441.72 per 100,000 population (95% UI: 229.59, 724.17). From 1990 to 2021, the age-standardized DALY rate increased in 16 countries, with Monaco showing the largest rise (AAPC = 0.85, 95% CI 0.84, 0.86). In contrast, the age-standardized DALY rate declined in 65 countries, with the most substantial reduction observed in South Sudan (AAPC = −140.80, 95% CI −157.95, −123.65. Additional File 1: Supplementary Table S5).

#### Global trends by age–gender group

**Fig. 2:**
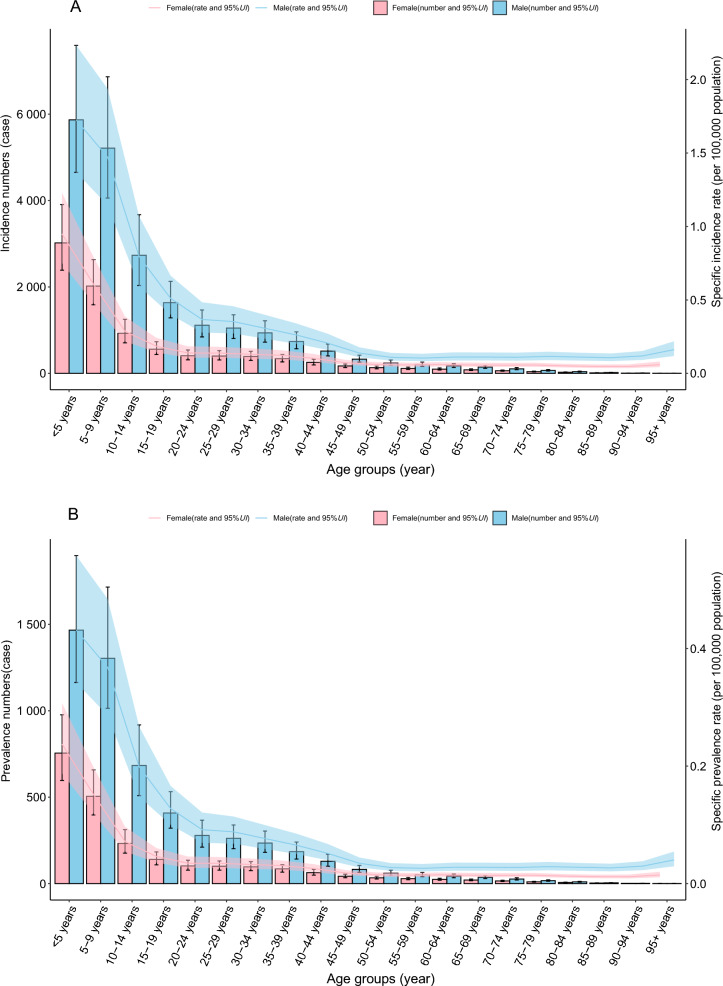

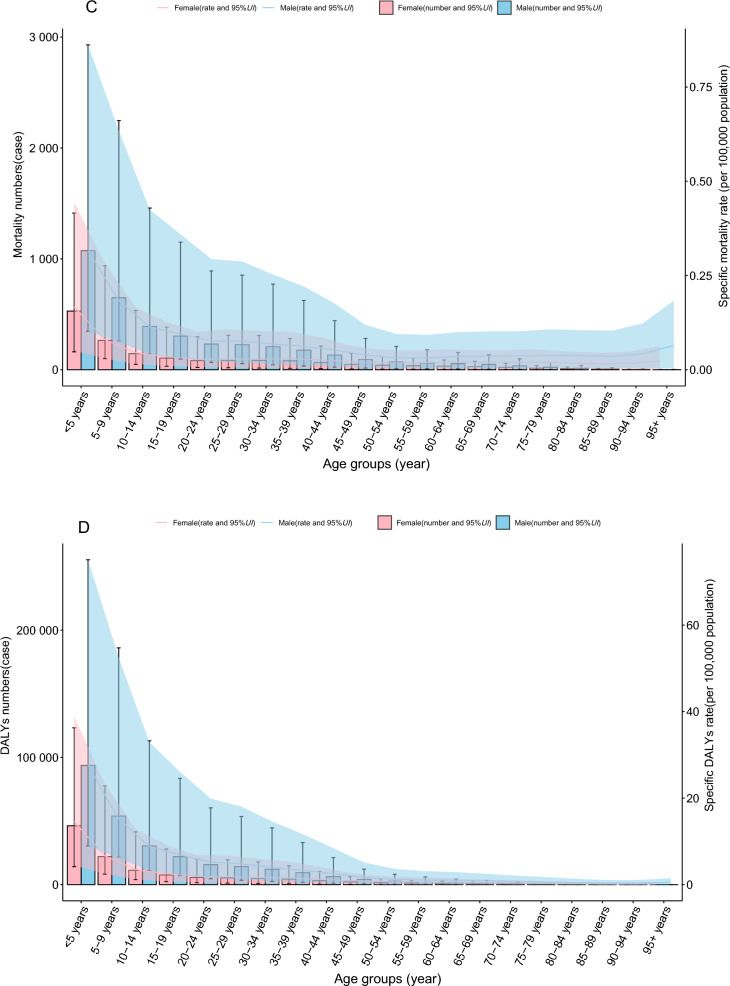
The specific rate of VL showed notable differences across age–gender distributions in 2021 year (**A** incidence; **B** prevalence; **C** mortality; **D** DALYs); each figure depicts the uncertainty intervals for sex-specific rates, with the blue-shaded area representing males and the red-shaded area representing females; *DALYs* disability-adjusted life years, *UI* uncertainty interval, *VL* visceral leishmaniasis

In 2021, the ASIR, ASPR, ASMR, and age-standardized DALY rate for VL exhibited a distinct L-shaped relationship with age. Across all age groups (in 5-year intervals), the ASIR and ASPR were consistently higher in males compared with females. However, no significant gender differences were observed in ASMR or the age-standardized DALY rate (Fig. 2A–D). In addition, no significant variation in case fatality rates was observed across different age groups (Additional File 1: Supplementary Table S6).

### The correlation between rate and SDI

**Fig. 3:**
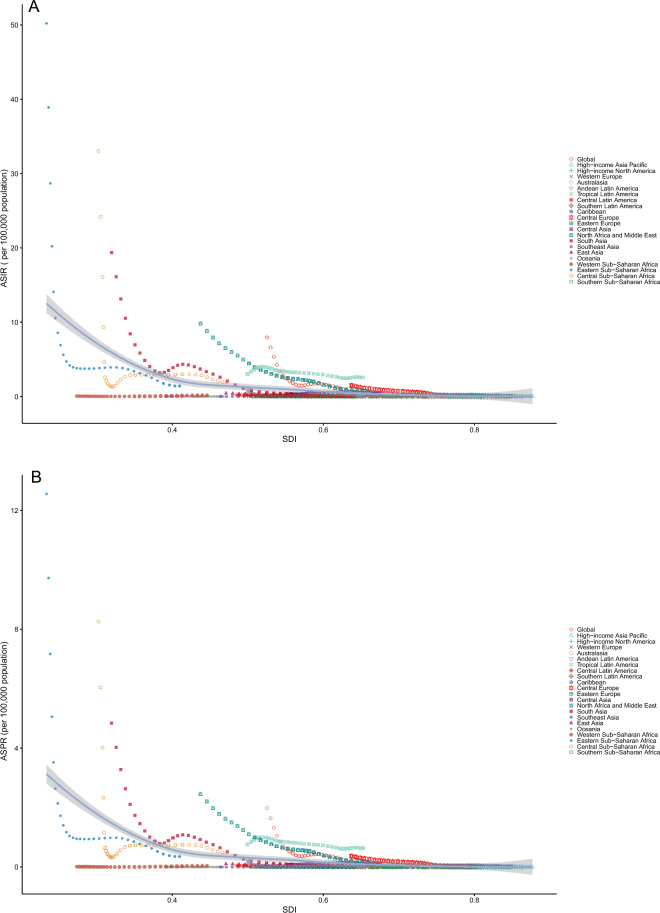

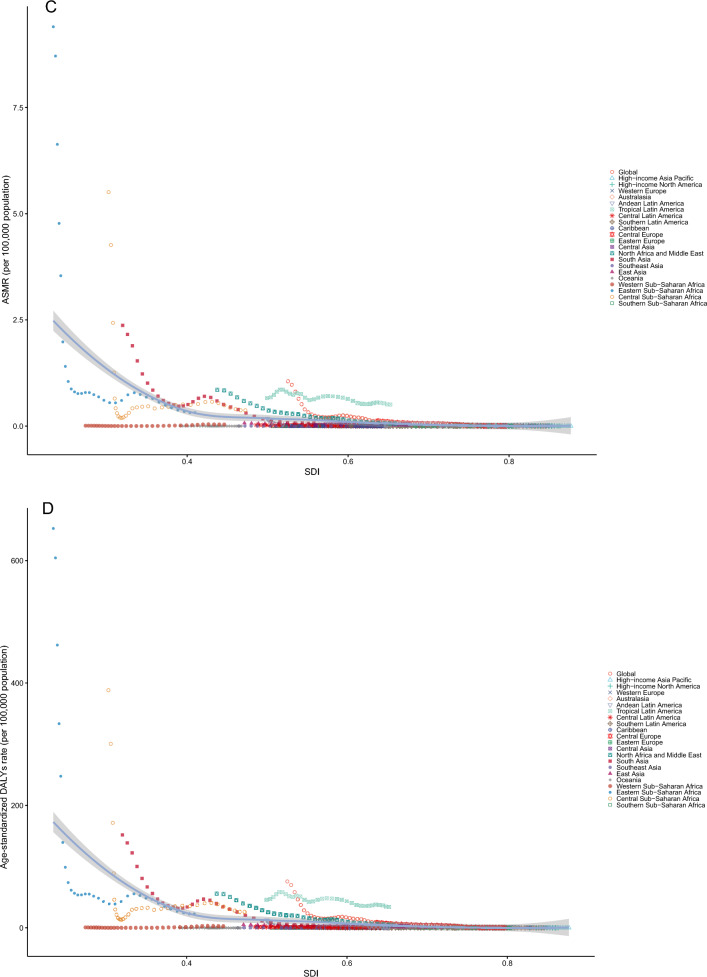
The association between the age-standardized rate of VL and the SDI in the global population and 21 geographical regions from 1990 to 2021(**A** ASIR; **B** ASPR; **C** ASMR; **D** age-standardized DALYs); in each of the four figures, the black curve depicts the relationship between the rate and SDI, with the shaded area denoting the 95% confidence interval; *ASIR* age-standardized incidence rate, *ASMR* age-standardized mortality rate, *ASPR* age-standardized prevalence rate, *DALYs* disability-adjusted life years, *SDI* sociodemographic index, *UI* uncertainty interval. *VL* visceral leishmaniasis

From 1990 to 2021, the ASIR (*r* = −0.483, *P* < 0.001), ASPR (*r* = −0.483, *P* < 0.001), ASMR (*r* = −0.511, *P* < 0.001), and age-standardized DALY rate (*r* = −0.514, *P* < 0.001) of VL and SDI showed a moderate negative correlation with SDI (Fig. 3A–D). In addition, the number of incidence cases (*r* = −0.467, *P* < 0.001), prevalence cases (*r *= −0.467, *P* < 0.001), deaths cases (*r* = −0.488, *P* < 0.001), and DALYs across person years (*r* = −0.497, *P* < 0.001) for these diseases exhibited a weak negative correlation with the SDI (Additional File 1: Supplementary Table S7).

## Discussion

This study provides a comprehensive analysis of the global trends and patterns in VL over the past 30 years, with a focus on key countries and regions. Between 1990 and 2021, the ASIR, ASMR, and age-standardized DALY rate for VL showed a consistent decline globally. Across all age groups, males exhibited higher ASIRs than females, with the highest ASMR observed in children under 5 years. However, during the same period, ASIR and ASMR increased in Central Asia and Western Sub-Saharan Africa. Projections for the next decade indicate a continued decline in the global burden of VL.

The study found that the ASMR for VL was highest among children under 5 years old. While an underdeveloped immune system may contribute to the higher mortality rate in children [33], the primary reason is likely the higher prevalence and incidence of VL in this age group. As a result, children naturally account for a larger proportion of total VL-related deaths. In fact, the overall threat of VL-related mortality does not significantly differ across age groups.

The study found a declining trend in the ASMR for VL from 1990 to 2021, likely driven by advancements in early diagnosis and treatment, expanded vector control efforts, increased public health investment, and greater health awareness in endemic regions [10, 34]. Improved diagnostic technologies and the widespread use of effective anti-leishmanial therapies, such as amphotericin B, have facilitated early intervention, reducing severe complications and mortality [10, 35]. Intensified vector control strategies, including insecticide application, environmental management, and health education, have contributed to lower transmission rates [35]. Strengthened healthcare infrastructure and international support have enhanced access to treatment and improved case management, particularly in resource-limited settings [6, 36]. In addition, rising awareness of VL transmission and symptoms has promoted earlier healthcare-seeking behavior, further mitigating disease severity and mortality [6, 36].

This study found that the ASIR of VL is consistently higher in males than in females across all age groups. Occupational and environmental exposure likely plays a key role, as men in endemic regions are more frequently engaged in outdoor activities, such as agriculture and construction, which increase contact with sandflies, the primary vectors of VL [37, 38]. Behavioral and lifestyle differences may further heighten risk, as men in these regions often participate more frequently in social or nighttime activities, coinciding with the peak biting activity of sandflies during evening and night hours [37, 38]. In addition, disparities in healthcare-seeking behavior and case reporting may influence observed incidence patterns. Cultural norms in certain regions may encourage men to seek medical care more readily, leading to higher case detection and reporting rates. In contrast, women may experience barriers such as economic constraints, domestic responsibilities, or sociocultural restrictions, which can delay or limit access to healthcare, potentially resulting in under-reporting of VL cases [38, 39]. These factors collectively contribute to the observed sex-based differences in VL incidence and underscore the need for targeted interventions to address gender-related disparities in disease exposure, healthcare access, and case detection.

Over the past 30 years, the burden of VL has risen rapidly in some countries and regions. Monaco, despite its small land area, high level of urbanization, excellent sanitation, and relatively low rate of canine infections, has historically not been a high-incidence area for the disease. However, in the past decade, the incidence of VL has increased significantly. As an international hub with frequent population movement, Monaco has seen a rise in imported cases, which may have contributed to local transmission [4, 40]. Meanwhile, South Sudan had the highest burden indicators for visceral VL in 2021, and the incidence, prevalence, or mortality rates of VL were also high in the Central African Republic, Monaco, Brazil, Djibouti, Chad, and Niger in 2021. To effectively control imported VL, a comprehensive strategy is essential. This includes strengthening case surveillance and early diagnosis, implementing health screenings and laboratory testing for travelers from endemic areas, and ensuring timely detection and management of imported cases [4, 40].

It is particularly important to emphasize that while AAPC provides insights into the relative trend of disease burden, it does not capture the absolute scale of the problem. Relying solely on AAPC may lead to misinterpretations, especially in countries where a high relative increase does not necessarily correspond to a significant public health threat. Absolute measures such as incidence, mortality, and DALYs are crucial for accurately assessing outbreak risks, optimizing healthcare resource allocation, and formulating effective control strategies. A comprehensive evaluation integrating both relative and absolute metrics is essential to ensure evidence-based decision-making in disease prevention and control [21, 27].

Building on the One Health framework, strategies for preventing and controlling leishmaniasis should integrate the interconnected health of humans, animals, and the environment [41–47]. A collaborative approach among public health, veterinary medicine, and environmental science is essential to establish integrated surveillance and early warning systems, enabling timely detection and response to outbreaks [40, 48, 49]. Efforts to reduce sandfly breeding habitats through targeted environmental interventions play a crucial role in minimizing disease transmission. Advancing the development and equitable distribution of vaccines can enhance immune protection in both human populations and animal reservoirs, further strengthening disease control efforts [40, 48]. Public awareness campaigns are vital to educate communities about leishmaniasis, its transmission pathways, and the importance of seeking early medical care [40, 48, 49]. Strengthening policy support and resource allocation, alongside fostering research, data sharing, and international collaboration, remains fundamental in addressing the challenges of VL control. The implementation of these comprehensive strategies can effectively reduce disease transmission and safeguard the health of humans, animals, and the environment [35, 40, 50, 51].

This study has limitations. The accuracy of disease burden estimates in the GBD 2021 framework depends on data quality and availability. Despite the use of advanced statistical models such as DisMod-MR 2.1 to address heterogeneity, disparities in data coverage, reporting mechanisms, classification standards, and surveillance quality persist across countries. Under-reporting remains a challenge in low- and middle-income countries owing to resource constraints, while developed nations with comprehensive surveillance may report lower burdens, introducing potential biases in global estimates [18–20]. In addition, reliance on model-based rather than direct observational data may lead to over- or underestimation of VL burden [18–20].

## Conclusions

The study systematically analyzed the global burden of VL and its trends from 1990 to 2021. The findings highlight that VL incidence is consistently higher in males, with children under 5 years identified as a high-risk group. While the ASIR and age-standardized DALY rate for VL have shown a declining trend, the disease remains a significant and under-recognized tropical health issue in Latin America, the Middle East, Africa, and South Asia. There is an urgent global need for more effective control measures to address the persistent threat of VL. Raising public health awareness and strengthening international collaboration in infectious disease response are essential for further reducing the burden of VL.

## Supplementary Information

Additional file 1. **Table S1**: The incidence cases of visceral leishmaniasis in 2021, and change trend of incidence cases were analyzed across GBD regions. **Table S2**: The prevalence cases of visceral leishmaniasis in 2021, and change trend of prevalence cases were analyzed across GBD regions. **Table S3**: The mortality case of visceral leishmaniasis in 2021, and change trend of mortality case were analyzed across GBD regions. **Table S4**: The DALYs case of visceral leishmaniasis in 2021, and change trend of DALYs case were analyzed across GBD regions. **Table S5**: The change trend of visceral leishmaniasis in 204 countries and territories of GBD from 1990 to 2021. **Table S6:** The case fatality rates of visceral leishmaniasis among age groups in 2021 year. **Table S7**: The correlation between the burden of visceral leishmaniasis and the SDI among 204 countries and territories in 2021year.

## Data Availability

No datasets were generated or analyzed during the current study.

## Abbreviations

AAPC: Average annual percentage change
ASIR: Age-standardized incidence rate
ASMR: Age-standardized mortality rate
ASPR: Age-standardized prevalence rate
ASR: Age-standardized rates
CI: Confidence interval
DALYs: Disability-adjusted life-years
EAPC: Estimated annual percentage change
GBD: Global Burden of Disease
ICD: International Classification of Diseases
SDI: Sociodemographic index
UI: Uncertainty interval
VL: Visceral leishmaniasis
WHO: World Health Organization
