# Supplementary material for: Global, regional, and national burden of visceral leishmaniasis, 1990–2021: findings from the global burden of disease study 2021
Source: Parasit Vectors. 2025 Apr 26;18:157. doi: 10.1186/s13071-025-06796-x (PMC12032768; doi:10.1186/s13071-025-06796-x)
Supplement: Supplementary file 1 — Additional file 1. Table S1: The incidence cases of visceral leishmaniasis in 2021, and change trend of incidence cases were analyzed across GBD regions. Table S2: The prevalence cases of visceral leishmaniasis in 2021, and change trend of prevalence cases were analyzed across GBD regions. Table S3: The mortality case of visceral leishmaniasis in 2021, and change trend of mortality case were analyzed across GBD regions. Table S4: The DALYs case of visceral leishmaniasis in 2021, and change trend of DALYs case were analyzed across GBD regions. Table S5: The change trend of visceral leishmaniasis in 204 countries and territories of GBD from 1990 to 2021. Table S6: The case fatality rates of visceral leishmaniasis among age groups in 2021 year. Table S7: The correlation between the burden of visceral leishmaniasis and the SDI among 204 countries and territories in 2021year. [file 13071_2025_6796_MOESM1_ESM.docx]

**Additional file 1**

**Supplementary materials**

Global, regional, and national burden of *Visceral leishmaniasis*, 1990–2021: Findings from the global burden of disease study 2021

**Items**

**Table S1**: The incidence cases of *Visceral leishmaniasis* in 2021, and change trend of incidence cases were analyzed across GBD regions.

**Table S2**: The prevalence cases of *Visceral leishmaniasis* in 2021, and change trend of prevalence cases were analyzed across GBD regions.

**Table S3**: The mortality case of *Visceral leishmaniasis* in 2021, and change trend of mortality case were analyzed across GBD regions.

**Table S4**: The DALYs case of *Visceral leishmaniasis* in 2021, and change trend of DALYs case were analyzed across GBD regions.

**Table S5**: The change trend of *Visceral leishmaniasis* in 204 countries and territories of GBD from 1990 to 2021.

**Table S6:** The case fatality rates of *Visceral leishmaniasis* among age groups in 2021 year.

**Table S7**: The correlation between the burden of *Visceral leishmaniasis* and the SDI among 204 countries and territories in 2021year.

Table S1: The incidence cases of *Visceral leishmaniasis* in 1990 and 2021, and change trend of incidence cases were analyzed across GBD regions.

| Location | *Visceral leishmaniasis.* Incidence cases (persons) . (95% *UI*).  1990 year | *Visceral leishmaniasis.* Incidence cases (persons) . (95% *UI*).  2021 year | *Visceral leishmaniasis.* Percentage change (95% *UI*). 1990－2021. | *Visceral leishmaniasis.* AAPC (95% *CI*). 1990－2021. |
| --- | --- | --- | --- | --- |
| Global | 466267(355564, 593218) | 30218(24630, 38446) | -93.5(-95.2,-90.6) | -13620.712(-14420.054,-12821.370) |
| East Asia | 5177.0(3722, 7251) | 844(636, 1106) | -83.7(-89.4,-74.9) | -142.063(-143.437,-140.689) |
| Southeast Asia | 39(20, 71) | 7(4, 13) | -81.2(-91.7,-52.9) | -1.037(-1.059,-1.016) |
| Central Asia | 211(149, 308) | 400(285, 584) | 89.7(15.9,209.3) | 5.804(5.373,6.235) |
| Central Europe | 1639(912, 3315) | 67(48, 95) | -95.9(-98.1,-91.6) | -50.654(-51.755,-49.554) |
| Western Europe | 736(573, 941) | 268(184, 392) | -63.6(-77.6,-42.9) | -14.951(-15.908,-13.993) |
| Southern Latin America | 38(17, 74) | 11(6, 19) | -71.5(-88.9,-27.5) | -0.857(-0.931,-0.784) |
| Caribbean | 10(2, 32) | 1(1, 3) | -90.4(-98.6,-38.8) | -0.285(-0.289,-0.281) |
| Andean Latin America | 391(69, 1298) | 30(6, 98) | -92.3(-99.0,-44.1) | -11.550(-11.726,-11.375) |
| Central Latin America | 355(243, 519) | 118(87, 159) | -66.7(-80.0,-47.6) | -7.445(-7.806,-7.084) |
| Tropical Latin America | 5209(3920, 6763) | 5052(2960, 8086) | -3.0(-45.9,65.0) | -6.418(-11.473,-1.364) |
| North Africa and Middle East | 41649(28045, 60871) | 4496(2758, 7060) | -89.2(-94.1,-80.5) | -1231.442(-1267.472,-1195.412) |
| South Asia | 257883(174260, 362623) | 6943(3845, 11623) | -97.3(-98.6,-94.8) | -8435.748(-8758.086,-8113.409) |
| Central Sub-Saharan Africa | 24141(8150, 60805) | 3379(1048, 8546) | -86.0(-96.8,-43.2) | -653.304(-677.711,-628.897) |
| Eastern Sub-Saharan Africa | 128667(78222, 200809) | 7469(5693, 9857) | -94.2(-96.5,-89.5) | -3912.261(-3988.642,-3835.880) |
| Western Sub-Saharan Africa | 123(72, 205) | 1134(449, 2536) | 820.1(225.9,2567.7) | 36.019(33.956,38.082) |
| High-middle SDI | 5744(3999, 8167) | 541(429, 673) | -90.6(-93.7,-85.5) | -168.002(-169.821,-166.183) |
| High SDI | 513(299, 884) | 45(27, 80) | -91.1(-95.8,-80.8) | -15.050(-15.222,-14.878) |
| Low-middle SDI | 101055.3(72395, 141562) | 10654(7970, 13885) | -89.5(-93.2,-83.4) | -3023.128(-3129.433,-2916.823) |
| Low SDI | 338856(245264, 451424.2) | 15756(11394, 22685) | -95.4(-97.0,-92.6) | -10572.502(-10803.268,-10341.735) |
| Middle SDI | 19956(16695, 23810) | 3209(2585, 4099) | -83.9(-87.8,-78.4) | -531.321(-540.402,-522.240) |

Notes: In the GBD 2021 database, 204 countries and territories are categorized into 21 geographical regions. However, no data on the ASIR of VL was available for Oceania, Eastern Europe, High-income North America, High-Income Asia Pacific, Australasia, or Southern Sub-Saharan Africa. Abbreviations: AAPC=Average Annual Percent Change. *CI*=Confidence interval. GBD=Global burden of disease. *UI*=Uncertainty interval. SDI=Socio-demographic index.

Table S2: The prevalence cases of *Visceral leishmaniasis* in 1990 and 2021, and change trend of prevalence cases were analyzed across GBD regions.

| Location | *Visceral leishmaniasis.* Prevalence cases (persons) . (95% *UI*).  1990 year. | *Visceral leishmaniasis.* Prevalence cases (persons) . (95% *UI*).  2021 year. | *Visceral leishmaniasis.* Percentage change (95% *UI*). 1990－2021. | *Visceral leishmaniasis.* AAPC (95% *CI*). 1990－2021. |
| --- | --- | --- | --- | --- |
| Global | 116567(88891, 148305) | 7554(6157, 96125) | -93.5(-95.2,-90.6) | -3591.114(-3677.586,-3504.641) |
| East Asia | 1294(931, 1813) | 211(159, 277) | -83.7(-89.4,-74.9) | -35.515(-35.859,-35.172) |
| Southeast Asia | 10(5, 18) | 2(1, 3) | -81.2(-91.7,-52.9) | -0.259(-0.265,-0.254) |
| Central Asia | 53(37,77) | 100(71, 146) | 89.7(15.9,209.3) | 1.451(1.343,1.559) |
| Central Europe | 410(228, 829) | 17(12, 24) | -95.9(-98.1,-91.6) | -12.664(-12.939,-12.389) |
| Western Europe | 184(143, 235) | 67(46, 98) | -63.6(-77.6,-42.9) | -3.738(-3.977,-3.498) |
| Southern Latin America | 10(4, 18) | 3(2, 5) | -71.5(-88.9,-27.5) | -0.214(-0.233,-0.196) |
| Caribbean | 3(1, 8) | 1(0, 1) | -90.4(-98.6,-38.8) | -0.070(-0.071,-0.068) |
| Andean Latin America | 98(17, 325) | 8(2, 25) | -92.3(-99.0,-44.1) | -2.892(-2.928,-2.855) |
| Central Latin America | 89(61, 130) | 30(22, 40) | -66.7(-80.0,-47.6) | -1.861(-1.952,-1.771) |
| Tropical Latin America | 1302(980,1691) | 1263(740, 2021) | -3.0(-45.9,65.0) | -1.606(-2.869,-0.342) |
| North Africa and Middle East | 10412(7011, 15218) | 1124(696, 1765) | -89.2(-94.1,-80.5) | -299.936(-308.563,-291.308) |
| South Asia | 64471(43565, 90656) | 1736(961, 2906 ) | -97.3(-98.6,-94.8) | -2118.498(-2218.806,-2018.190) |
| Central Sub-Saharan Africa | 6035(2038, 15201) | 845(262, 2137) | -86.0(-96.8,-43.2) | -166.879(-171.869,-161.890) |
| Eastern Sub-Saharan Africa | 32167(19556, 50202) | 1867(1423, 2464) | -94.2(-96.5,-89.5) | -978.076(-996.056,-960.096) |
| Western Sub-Saharan Africa | 31(18, 51) | 284(112, 634) | 820.1(225.9,2567.7) | 9.004(8.489,9.520) |
| High-middle SDI | 1436(1000, 2042) | 135(107, 168) | -90.6(-93.7,-85.5) | -42.274(-42.637,-41.911) |
| High SDI | 128(75, 221) | 12(7, 20) | -91.1(-95.8,-80.8) | -3.755(-3.806,-3.703) |
| Low-middle SDI | 25264(18099, 35390) | 2664(1993, 3471) | -89.5(-93.2,-83.4) | -761.145(-789.067,-733.223) |
| Low SDI | 84714(61316, 112856) | 3939(2849, 5671) | -95.4(-97.0,-92.6) | -2643.136(-2700.828,-2585.444) |
| Middle SDI | 4989(4174, 5953) | 802(646,1025) | -83.9(-87.8,-78.4) | -132.828(-135.099,-130.558) |

Notes: In the GBD 2021 database, 204 countries and territories are categorized into 21 geographical regions. However, no data on the ASIR of VL was available for Oceania, Eastern Europe, High-income North America, High-Income Asia Pacific, Australasia, or Southern Sub-Saharan Africa. Abbreviations: AAPC=Average Annual Percent Change. *CI*=Confidence interval. GBD=Global burden of disease. *UI*=Uncertainty interval. SDI=Socio-demographic index.

Table S3: The mortality case of *Visceral leishmaniasis* in 1990 and 2021, and change trend of mortality case were analyzed across GBD regions.

| Location | *Visceral leishmaniasis.* Death cases (persons)  (95% *UI*).  1990 year | *Visceral leishmaniasis.* Death cases (persons)  (95% *UI*).  2021 year | *Visceral leishmaniasis.* Percentage change (95% *UI*). 1990－2021. | *Visceral leishmaniasis* AAPC (95% *CI*). 1990－2021. |
| --- | --- | --- | --- | --- |
| Global | 60245(20096,193319) | 5482(1610, 17781) | -90.9(-92.7,-88.5) | -1803.481(-1885.589,-1721.373) |
| East Asia | 864(1, 4752) | 144(0, 759) | -83.3(-85.8,-74.6) | -23.325(-23.757,-22.893) |
| Southeast Asia | 8(1, 34) | 2(0, 7) | -76.6(-81.3,-60.6) | -0.186(-0.196,-0.176) |
| Central Asia | 18(1, 163) | 36(0, 308) | 98.0(85.9,148.4) | 0.601(0.539,0.662) |
| Central Europe | 145(0, 1310) | 6(0, 49) | -96.0(-96.3,-94.6) | -4.415(-4.495,-4.334) |
| Western Europe | 83(21, 495) | 25.8(6.5,142.1) | -68.9(-75.1,-59.8) | -1.969(-2.245,-1.692) |
| Southern Latin America | 3(0, 29) | 1(0, 9) | -69.3(-70.4,-64.9) | -0.073(-0.079,-0.067) |
| Caribbean | 2(0, 8) | 1(1, 2) | -88.8(-90.8,-83.1) | -0.049(-0.050,-0.048) |
| Andean Latin America | 76(1, 390) | 7(1, 31) | -91.6(-93.6,-87.7) | -2.233(-2.265,-2.200) |
| Central Latin America | 45(0, 310) | 17(1,105) | -63.1(-68.9,-44.7) | -0.882(-0.983,-0.782) |
| Tropical Latin America | 1073(1, 4134) | 1077(1, 3748) | 0.5(-11.7,42.2) | 2.173(-2.062,6.409) |
| North Africa and Middle East | 3223(1, 27202) | 411(1, 3547) | -87.3(-90.3,-84.1) | -97.821(-100.986,-94.656) |
| South Asia | 27849(5, 126974) | 1252(1, 6490) | -95.5(-96.9,-94.6) | -865.626(-911.106,-820.145) |
| Central Sub-Saharan Africa | 3904(2040, 6208) | 613(312, 998) | -84.3(-87.0,-81.1) | -109.289(-113.227,-105.350) |
| Eastern Sub-Saharan Africa | 22930(15416, 32154) | 1639(969, 2492) | -92.9(-94.7,-90.7) | -731.082(-757.798,-704.366) |
| Western Sub-Saharan Africa | 25(13, 40) | 253(132, 418) | 930.2(728.2,1192.4) | 7.382(7.169,7.594) |
| High-middle SDI | 537(13, 4613) | 60(7, 354) | -88.9(-92.3,-45.9) | -16.508(-17.339,-15.677) |
| High SDI | 55(6, 414) | 5(1, 35) | -91.6(-99.3,-88.5) | -1.724(-2.035,-1.413) |
| Low-middle SDI | 12690(2073, 52418) | 1729(242, 7482) | -86.4(-90.0,-83.2) | -352.336(-367.498,-337.174) |
| Low SDI | 44596(17669, 122184) | 3114(1306, 7359) | -93.0(-95.0,-88.9) | -1411.549(-1449.852,-1373.245) |
| Middle SDI | 2346(1, 14066) | 574(1, 2620) | -75.6(-81.5,-28.2) | -61.746(-64.114,-59.379) |

Notes: In the GBD 2021 database, 204 countries and territories are categorized into 21 geographical regions. However, no data on the ASIR of VL was available for Oceania, Eastern Europe, High-income North America, High-Income Asia Pacific, Australasia, or Southern Sub-Saharan Africa. Abbreviations: AAPC=Average Annual Percent Change. *CI*=Confidence interval. GBD=Global burden of disease. *UI*=Uncertainty interval. SDI=Socio-demographic index.

Table S4: The DALYs case of *Visceral leishmaniasis* in 1990 and 2021, and change trend of DALYs case were analyzed across GBD regions.

| Location | *Visceral leishmaniasis* DALYs cases. (persons)  (95% *UI*).  1990 year. | *Visceral leishmaniasis* DALYs cases. (persons)  (95% *UI*).  2021 year. | *Visceral leishmaniasis.* Percentage change (95% *UI*). 1990－2021. | *Visceral leishmaniasis.* AAPC (95% *CI*). 1990－2021. |
| --- | --- | --- | --- | --- |
| Global | 4475429(1552457,14360034) | 388212(121442, 1248115) | -91.3(-93.1, -88.6) | -134573.222(-140544.383, -128602.060) |
| East Asia | 61637(104, 346232) | 8548(16.4,47661) | -86.1(-88.3, -80.1) | -1722.711(-1751.807, -1693.616) |
| Southeast Asia | 527(1, 2433) | 103(1, 445) | -80.4(-84.3,-70.2) | -13.390(-14.041,-12.740) |
| Central Asia | 1266 (3, 11782) | 2350(6, 20957) | 85.6(55.8,150.9) | 36.014(32.562, 39.467) |
| Central Europe | 9181(23, 87849) | 320(1, 2921) | -96.5(-97.2,-94.6) | -281.539(-286.487, -276.592) |
| Western Europe | 4538(939, 31505) | 1375(381, 8071) | -69.7(-74.8,-54.9) | -104.431(-117.316, -91.545) |
| Southern Latin America | 195(1, 2026) | 55(1, 569) | -71.6(-81.7,-50.3) | -5.075(-5.454,-4.696) |
| Caribbean | 123(1,608) | 13(0, 59) | -89.7(-93.2,-81.4) | -3.495(-3.563,-3.427) |
| Andean Latin America | 5754(5, 30009) | 449(1, 2262) | -92.2(-94.9,-86.9) | -169.718(-172.138,-167.298) |
| Central Latin America | 3312(6, 23366) | 1088(2, 7263) | -67.2(-74.9,-54.2) | -68.564(-76.075,-61.054) |
| Tropical Latin America | 77942(105, 306755) | 67902(112, 246283) | -12.9(-23.5,22.4) | -181.503(-465.963,102.957) |
| North Africa and Middle East | 236783(655, 2033680) | 29493(72, 260000) | -87.5(-91.7,-83.7) | -7200.031(-7429.571,-6970.491) |
| South Asia | 1992526(4402, 9275132) | 84667(128, 452018) | -95.8(-97.7,-94.8) | -62116.961(-65289.652,-58944.271) |
| Central Sub-Saharan Africa | 303296(159033, 484788) | 46308(23293, 75967) | -84.7(-87.3,-81.7) | -8530.645(-8834.320,-8226.970) |
| Eastern Sub-Saharan Africa | 1776407.6(1187133, 2523426) | 125548(73443, 191461) | -92.9(-94.9,-90.7) | -56690.173(-58784.389,-54595.957) |
| Southern Sub-Saharan Africa | NA | NA | NA | NA |
| Western Sub-Saharan Africa | 1941(1048. 53177) | 19995(10459, 32858) | 930.2(728.4,1187.6) | 583.661(565.073,602.248) |
| High-middle SDI | 36283(958,323552) | 3434(399, 21848) | -90.5(-93.2,-55.8) | -1135.973(-1193.184,-1078.763) |
| High SDI | 3191(78, 29097) | 256(6, 2134) | -92.0(-95.7,-88.5) | -93.283(-98.568,-87.999) |
| Low-middle SDI | 941617(162459, 3915809) | 118954(18567, 527893) | -87.4(-90.2,-84.5) | -26421.749(-27525.430,-25318.067) |
| Low SDI | 3326033(1359812, 9038028) | 230448(99374, 534936) | -93.1(-95.1,-88.9) | -105096.852(-107783.697,-102410.007) |
| Middle SDI | 166751(394, 1023617) | 34976(89, 170137) | -79.0(-83.5,-60.3) | -4571.303(-4740.820,-4401.787) |

Notes: In the GBD 2021 database, 204 countries and territories are categorized into 21 geographical regions. However, no data on the ASIR of VL was available for Oceania, Eastern Europe, High-income North America, High-Income Asia Pacific, Australasia, or Southern Sub-Saharan Africa. Abbreviations: AAPC=Average Annual Percent Change. *CI*=Confidence interval. GBD=Global burden of disease. *UI*=Uncertainty interval. SDI=Socio-demographic index.

Table S5: The change trend of *Visceral leishmaniasis* in 204 countries and territories of GBD from 1990 to 2021.

| Feature | Rank | Year | Index | Rate | Nations | Value (95% *UI*) |
| --- | --- | --- | --- | --- | --- | --- |
| Ascending | 1 | 1990-2021 | ASIR | Rate | Monaco | 0.153(0.149,0.157) |
| Ascending | 2 | 1990-2021 | ASIR | Rate | Georgia | 0.057(0.038,0.076) |
| Ascending | 3 | 1990-2021 | ASIR | Rate | Niger | 0.054(0.050,0.058) |
| Ascending | 4 | 1990-2021 | ASIR | Rate | Chad | 0.029(0.028,0.031) |
| Ascending | 5 | 1990-2021 | ASIR | Rate | Armenia | 0.018(0.018,0.019) |
| Descending | 1 | 1990-2021 | ASIR | Rate | Somalia | -10.696(-10.846,-10.546) |
| Descending | 2 | 1990-2021 | ASIR | Rate | Ethiopia | -3.141(-3.186,-3.096) |
| Descending | 3 | 1990-2021 | ASIR | Rate | South Sudan | -2.817(-3.016,-2.619) |
| Descending | 4 | 1990-2021 | ASIR | Rate | Zambia | -2.688(-2.722,-2.655) |
| Descending | 5 | 1990-2021 | ASIR | Rate | Djibouti | -2.205(-2.247,-2.163) |
| Ascending | 1 | 1990-2021 | ASPR | Rate | Monaco | 0.038(0.038,0.039) |
| Ascending | 2 | 1990-2021 | ASPR | Rate | Georgia | 0.014(0.009,0.019) |
| Ascending | 3 | 1990-2021 | ASPR | Rate | Niger | 0.012(0.011,0.012) |
| Ascending | 4 | 1990-2021 | ASPR | Rate | Chad | 0.007(0.007,0.008) |
| Ascending | 5 | 1990-2021 | ASPR | Rate | Armenia | 0.005(0.004,0.005) |
| Descending | 1 | 1990-2021 | ASPR | Rate | Somalia | -2.672(-2.712,-2.632) |
| Descending | 2 | 1990-2021 | ASPR | Rate | Ethiopia | -0.785(-0.797,-0.774) |
| Descending | 3 | 1990-2021 | ASPR | Rate | South Sudan | -0.704(-0.754,-0.655) |
| Descending | 4 | 1990-2021 | ASPR | Rate | Zambia | -0.672(-0.681,-0.664) |
| Descending | 5 | 1990-2021 | ASPR | Rate | Djibouti | -0.545(-0.556,-0.535) |
| Ascending | 1 | 1990-2021 | ASMR | Rate | Monaco | 0.012(0.012,0.012) |
| Ascending | 2 | 1990-2021 | ASMR | Rate | Niger | 0.010(0.009,0.010) |
| Ascending | 3 | 1990-2021 | ASMR | Rate | Chad | 0.007(0.007,0.008) |
| Ascending | 4 | 1990-2021 | ASMR | Rate | Georgia | 0.004(0.003,0.005) |
| Ascending | 5 | 1990-2021 | ASMR | Rate | Senegal | 0.003(0.003,0.003) |
| Descending | 1 | 1990-2021 | ASMR | Rate | South Sudan | -2.015(-2.274,-1.757) |
| Descending | 2 | 1990-2021 | ASMR | Rate | Somalia | -1.573(-1.634,-1.512) |
| Descending | 3 | 1990-2021 | ASMR | Rate | Zambia | -0.586(-0.611,-0.562) |
| Descending | 4 | 1990-2021 | ASMR | Rate | Ethiopia | -0.558(-0.570,-0.546) |
| Descending | 5 | 1990-2021 | ASMR | Rate | Djibouti | -0.494(-0.509,-0.479) |
| Ascending | 1 | 1990-2021 | Age-standardized DALY rate | Rate | Monaco | 0.849(0.836,0.861) |
| Ascending | 2 | 1990-2021 | Age-standardized DALY rate | Rate | Niger | 0.692(0.661,0.724) |
| Ascending | 3 | 1990-2021 | Age-standardized DALY rate | Rate | Chad | 0.531(0.512,0.550) |
| Ascending | 4 | 1990-2021 | Age-standardized DALY rate | Rate | Georgia | 0.254(0.172,0.336) |
| Ascending | 5 | 1990-2021 | Age-standardized DALY rate | Rate | Senegal | 0.208(0.194,0.221) |
| Descending | 1 | 1990-2021 | Age-standardized DALY rate | Rate | South Sudan | -140.798(-157.947,-123.649) |
| Descending | 2 | 1990-2021 | Age-standardized DALY rate | Rate | Somalia | -106.561(-110.733,-102.388) |
| Descending | 3 | 1990-2021 | Age-standardized DALY rate | Rate | Zambia | -40.510(-42.260,-38.760) |
| Descending | 4 | 1990-2021 | Age-standardized DALY rate | Rate | Ethiopia | -38.392(-39.217,-37.567) |
| Descending | 5 | 1990-2021 | Age-standardized DALY rate | Rate | Djibouti | -35.285(-36.390,-34.179) |
| High | 1 | 2021 | ASIR | Rate | South Sudan | 27.13(15.47, 45.990) |
| High | 2 | 2021 | ASIR | Rate | Sudan | 6.790(3.570, 11.740) |
| High | 3 | 2021 | ASIR | Rate | Central African Republic | 6.290(1.140, 19.700) |
| High | 4 | 2021 | ASIR | Rate | Monaco | 4.981(0.980, 15.430) |
| High | 5 | 2021 | ASIR | Rate | Djibouti | 3.672(1.641, 7.052) |
| Low | 1 | 2021 | ASIR | Rate | Mexico | 0.010(0.001, 0.011) |
| Low | 2 | 2021 | ASIR | Rate | Thailand | 0.010(0.010, 0.020) |
| Low | 3 | 2021 | ASIR | Rate | Sri Lanka | 0.010(0.001, 0.030) |
| Low | 4 | 2021 | ASIR | Rate | Pakistan | 0.010(0.00, 0.030) |
| Low | 5 | 2021 | ASIR | Rate | Nigeria | 0.020(0.010, 0.040) |
| High | 1 | 2021 | ASPR | Rate | South Sudan | 6.780(3.87, 11.500) |
| High | 2 | 2021 | ASPR | Rate | Sudan | 1.700(0.890, 2.930) |
| High | 3 | 2021 | ASPR | Rate | Central African Republic | 1.570(0.280, 4.920) |
| High | 4 | 2021 | ASPR | Rate | Monaco | 1.240(0.250, 3.860) |
| High | 5 | 2021 | ASPR | Rate | Djibouti | 0.920(0.410, 1.760) |
| Low | 1 | 2021 | ASPR | Rate | Mexico | 0.010(0.001, 0.020) |
| Low | 2 | 2021 | ASPR | Rate | Thailand | 0.010(0.001, 0.020) |
| Low | 3 | 2021 | ASPR | Rate | Sri Lanka | 0.010(0.001, 0.020) |
| Low | 4 | 2021 | ASPR | Rate | Pakistan | 0.010(0.001, 0.020) |
| Low | 5 | 2021 | ASPR | Rate | Nigeria | 0.010(0.001, 0.020) |
| High | 1 | 2021 | ASMR | Rate | South Sudan | 6.130(3.180, 9.920) |
| High | 2 | 2021 | ASMR | Rate | Central African Republic | 1.190(0.600, 2.010) |
| High | 3 | 2021 | ASMR | Rate | Djibouti | 1.110(00.56, 1.900) |
| High | 4 | 2021 | ASMR | Rate | Sudan | 0.680(0.001, 5.440) |
| High | 5 | 2021 | ASMR | Rate | Somalia | 0.530(0.260, 0.910) |
| Low | 1 | 2021 | ASMR | Rate | Mexican | 0.010(0.001, 0.020) |
| Low | 2 | 2021 | ASMR | Rate | Romania | 0.010(0.001, 0.020) |
| Low | 3 | 2021 | ASMR | Rate | Israel | 0.010(0.001, 0.020) |
| Low | 4 | 2021 | ASMR | Rate | Saudi Arabia | 0.010(0.001, 0.020) |
| Low | 5 | 2021 | ASMR | Rate | Jordan | 0.010(0.001, 0.020) |
| High | 1 | 2021 | Age-standardized DALY rate | Rate | South Sudan | 441.72(229.59, 724.17) |
| High | 2 | 2021 | Age-standardized DALY rate | Rate | Central African Republic | 83.940(42.070, 143.430) |
| High | 3 | 2021 | Age-standardized DALY rate | Rate | Djibouti | 81.700(41.250, 138.930) |
| High | 4 | 2021 | Age-standardized DALY rate | Rate | Sudan | 45.230(0.100, 384.070) |
| High | 5 | 2021 | Age-standardized DALY rate | Rate | Eritrea | 38.390(18.95, 63.29) |
| Low | 1 | 2021 | Age-standardized DALY rate | Rate | Mexican | 0.060(0.001, 0.440) |
| Low | 2 | 2021 | Age-standardized DALY rate | Rate | Nigeria | 0.350(0.170, 0.600) |
| Low | 3 | 2021 | Age-standardized DALY rate | Rate | Thailand | 0.160(0.001, 0.690) |
| Low | 4 | 2021 | Age-standardized DALY rate | Rate | Sri Lanka | 0.160(0.001, 0.730) |
| Low | 5 | 2021 | Age-standardized DALY rate | Rate | Republic of C ô te d'Ivoire | 0.540(0.260, 0.910) |

Abbreviations: ASIR=Age-standardized incidence rate. ASMR=age-standardized mortality rate. ASPR=Age-standardized prevalence rate. DALYs= Disability-adjusted life years. GBD=Global burden of disease. *UI*=Uncertainty interval.

**Table S6:** The case fatality rates of *Visceral leishmaniasis* among age groups in 2021 year.

| Age group | death | incidence | CFR(95%CI) |
| --- | --- | --- | --- |
| <5 years | 0.244 | 1.350 | 0.18(0.003, 0.358) |
| 5-9 years | 0.133 | 1.053 | 0.127(0, 0.28) |
| 25-29 years | 0.053 | 0.247 | 0.215(0.025, 0.405) |
| 30-34 years | 0.048 | 0.219 | 0.22(0.029, 0.412) |
| 35-39 years | 0.046 | 0.193 | 0.238(0.041, 0.435) |
| 40-44 years | 0.039 | 0.154 | 0.255(0.054, 0.457) |
| 45-49 years | 0.030 | 0.106 | 0.284(0.075, 0.492) |
| 50-54 years | 0.025 | 0.084 | 0.293(0.083, 0.504) |
| 55-59 years | 0.025 | 0.080 | 0.307(0.094, 0.521) |
| 60-64 years | 0.027 | 0.085 | 0.323(0.107, 0.539) |
| 65-69 years | 0.028 | 0.083 | 0.331(0.114, 0.549) |
| 70-74 years | 0.028 | 0.081 | 0.341(0.122, 0.56) |
| 75-79 years | 0.028 | 0.083 | 0.337(0.119, 0.556) |
| 80-84 years | 0.026 | 0.076 | 0.341(0.122, 0.56) |
| 85-89 years | 0.024 | 0.070 | 0.339(0.121, 0.558) |
| 90-94 years | 0.025 | 0.070 | 0.362(0.14, 0.585) |
| 95+ years | 0.034 | 0.088 | 0.391(0.165, 0.616) |
| 75-84 years | 0.027 | 0.081 | 0.339(0.12, 0.557) |

Abbreviations: CFR= case fatality rates; CI=confidence intervals.The case fatality rates were comparable across all age groups, with overlapping confidence intervals (CIs), indicating no significant difference.

Table S7: The correlation between the burden of *Visceral leishmaniasis* and the SDI among 204 countries and territories in 2021 year.

| Index | *r* | P |
| --- | --- | --- |
| Incidence cases | -0.2799 | <0.001 |
| Prevalence cases | -0.2799 | <0.001 |
| Death cases | -0.2947 | <0.001 |
| DALYs cases | -0.2996, | <0.001 |

Abbreviations: DALYs=disability-adjusted life years; SDI=sociodemographic index.
